# Photo‐Imprinting of the Helical Organization in Liquid‐Crystal Networks Using Achiral Monomers and Circularly Polarized Light

**DOI:** 10.1002/anie.202200839

**Published:** 2022-02-18

**Authors:** Hirotoshi Sakaino, Dirk J. Broer, Stefan C. J. Meskers, E. W. Meijer, Ghislaine Vantomme

**Affiliations:** ^1^ Institute for Complex Molecular Systems Laboratory of Macromolecular and Organic Chemistry Eindhoven University of Technology P.O. Box 513 5600 MB Eindhoven The Netherlands; ^2^ Electronic & Imaging Materials Research Laboratories Toray Industries, Inc. 3-1-2 Sonoyama Otsu Shiga 520-0842 Japan; ^3^ Institute for Complex Molecular Systems Laboratory of Stimuli-Responsive Functional Materials and Devices Eindhoven University of Technology P.O. Box 513 5600 MB Eindhoven The Netherlands; ^4^ Institute for Complex Molecular Systems Molecular Materials and Nanosystems Eindhoven University of Technology P.O. Box 513 5600 MB Eindhoven The Netherlands

**Keywords:** Chirality, Cholesteric Liquid Crystals, Circularly Polarized Light, Mesogens, Nanostructures

## Abstract

Control over molecular motion is facilitated in materials with highly ordered nanoscale structures. Here we report on the fabrication of cholesteric liquid‐crystal networks by circularly polarized light irradiation, without the need for chiral dopant or plasticizer. The polymer network is obtained by photopolymerization of a smectic achiral diacrylate mesogen consisting of an azobenzene core and discrete oligodimethylsiloxane tails. The synchronous helical photoalignment and photopolymerization originate from the cooperative movement of the mesogens ordered in well‐defined responsive structures, together with the flexibility of the oligodimethylsiloxane blocks. The resulting thin films show excellent thermal stability and light‐induced memory features with reversible responses. Additionally, we demonstrate the fabrication of photo‐patterned films of liquid‐crystal networks with opposite helical senses. These findings provide a new method to make light‐controllable chiroptical materials with exciting applications in optics and photonics.

## Graphical abstract

A method has been developed to make cholesteric liquid‐crystal networks from achiral monomers by irradiation with circularly polarized light. This approach allows simultaneous control over the photoalignment of the mesogens and the photopolymerization of the network.

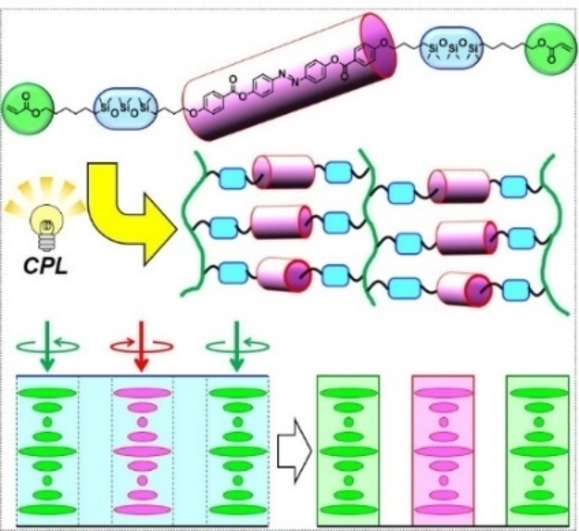

Cholesteric liquid‐crystal (LC) materials are at the forefront of the development of optoelectronics and photonics.[^1^, ^2^, ^3^] In the fabrication of cholesteric LC materials, the ability to tune the helical structure offers remarkable control over the material properties. Methods to prepare cholesteric LC materials generally involve doping the nematic matrix with chiral components.^[1]^ For example, in liquid crystal networks (LCNs),^[4]^ minute amounts of chiral dopants are mixed with diacrylate mesogens and a photoinitiator. After the formation of the cholesteric structure, the sample is irradiated with light to initiate photopolymerization, locking the cholesteric LC phase into the network. However, the adaptivity of these LC materials is limited due to the permanent embedding of the chiral dopant combined with the structure and the cross‐linking density of the polymer network.

As an alternative to the use of chiral dopant, the photoalignment by irradiation with circularly polarized light (CPL) is another well‐developed approach to imprint chirality into materials.[^5^, ^6^, ^7^, ^8^, ^9^, ^10^, ^11^] This method offers the possibility to generate spatially‐resolved patterns aligned in multidomains.[^12^, ^13^] Thereof, the photoalignment is mediated by the embedding of photoactive units into the ordered hierarchical assemblies of LC polymers.^[7]^ Upon irradiation with polarized light, photoactive units such as azobenzenes align their transition dipole moment perpendicular to the electric vector *E* of the incoming light by continuous trans‐cis‐trans isomerization. This method has been extensively studied to align LC polymers with photoactive units in the side‐chains because of their large degree of freedom, which facilitates reorientation of the mobile chromophores by light.[^14^, ^15^, ^16^] However, it remains to be demonstrated that this method can produce cholesteric LCNs. The challenge lies in the synchronization between the kinetics of CPL alignment and immobilization of the mesogens by photopolymerization. Indeed, it requires to control the balance between the stability of the photoalignment of low‐molecular‐weight LCs and the mobility of the cross‐linked polymer chains. Upon polymerization, increase in cross‐link density improves thermal stability of photoalignment due to the reduced mobility of the polymer chains, but at the same time precludes the helical photo‐ordering of the photosensitive fragments. Design strategies to control molecular motion in the fabrication of LCN are lacking.

Recently, organized nanoscale structures have been exploited to translate molecular transformation of photoswitches into controlled macroscopic function.[^17^, ^18^, ^19^, ^20^, ^21^] To reach perfectly nano‐ordered structures, block co‐oligomers with one block consisting of oligodimethylsiloxane (*o*DMS) of discrete length have been exploited.[^22^, ^23^] They assemble into well‐defined phase‐segregated structures with sub‐10 nm feature sizes. The role of discrete siloxanes provides both the well‐defined ordering of the photoswitches by phase separation and the flexibility of the polymer chains to improve segmental mobility.[^24^, ^25^] Hence, we envision that nano‐segregated mesogens combining azobenzenes and discrete oDMS will allow the alliance of nanoscale properties and flexibility in LC materials[^26^, ^27^, ^28^, ^29^] and could offer a strategy to prepare cholesteric LCN by CPL irradiation.

Here, we explore the imprinting of chirality in LCN by CPL irradiation using an achiral monomer **RM‐AzoSi_3_** (Figure 1). We present a reactive photo‐responsive mesogen able to align into helical structures under CPL and simultaneously photopolymerize into LCN. The mesogen is composed of an azobenzene core decorated with discrete siloxane tails and terminated with polymerizable acrylate units. We study in‐depth the morphological properties of the material and its thermo‐ and photo‐stability. We finally demonstrate photo‐patterning of opposite helical structures on the same LCN film.

**Figure 1:**
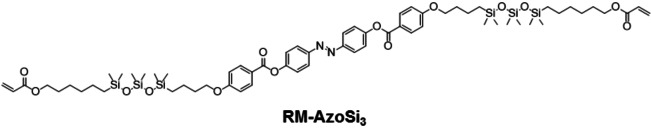
Chemical structure of **RM‐AzoSi_3_**.

The reactive azobenzene‐siloxane triblock mesogen **RM‐AzoSi_3_** was synthesized in four steps by coupling of the symmetric olefin‐terminated azobenzene and asymmetric alcohol‐functionalized *o*DMS‐monohydride (Figures S1–S12). The azobenzene central block was linked to the alcohol‐functionalized *o*DMS‐monohydride via a platinum catalyzed hydrosilylation reaction. And the triblock molecule obtained was derivatized with acryloyl end‐groups. **RM‐AzoSi_3_** was obtained as a viscous orange liquid at 25 °C. The thermal properties of **RM‐AzoSi_3_** showed three major exothermic transitions around 55 °C, 30 °C and −16 °C and a fourth, much weaker exotherm at 13 °C (Figure 2a). Going through these transitions in POM, **RM‐AzoSi_3_** changed from an isotropic melt to a birefringent viscous liquid characteristic of smectic LC phases (Figure S13).

**Figure 2:**
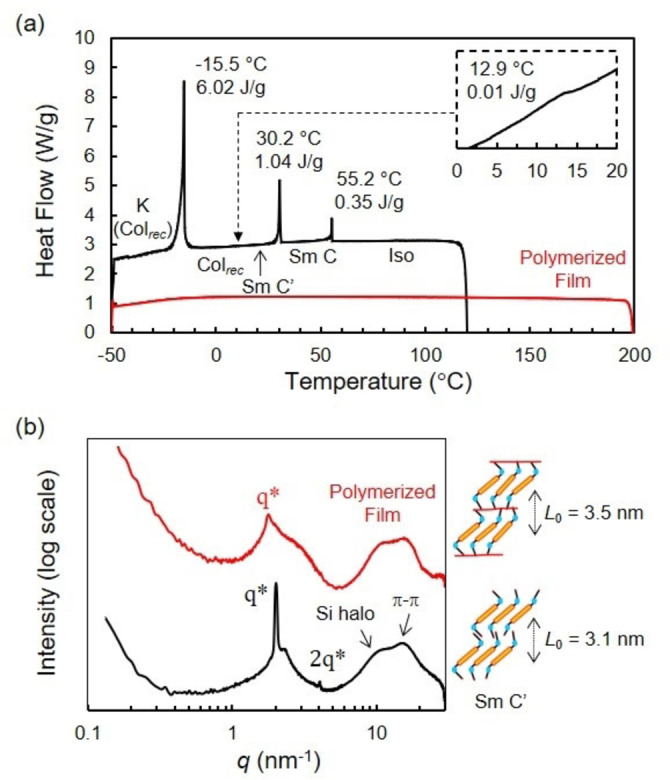
a) DSC traces (cooling run) and b) 1D transmission scattering profiles of **RM‐AzoSi_3_** before (black trace) and after (red trace) photopolymerization at 25 °C.

To analyze the conformation of the azobenzenes in the LC state, UV experiments were performed. In dichloromethane solution, **RM‐AzoSi_3_** exhibits a maximum absorption at 331 nm corresponding to the π–π* absorption band of the azobenzene trans isomers (Figure S14). On spin‐cast thin films, the maximum absorption of **RM‐AzoSi_3_** red‐shifts to 337 nm, indicating the formation of a *J*‐type aggregated structure of the azobenzenes in the lamellar morphology. Variable temperature small angle X‐ray scattering (SAXS) measurements were conducted to confirm the rich morphological properties of **RM‐AzoSi_3_**. Four distinct morphologies with domain spacings sub‐10 nm were characterized: a smectic C (Sm C) phase at 40 °C, a modulated or deformed smectic C (Sm C′) phase[^30^, ^31^] at 20 °C, a rectangular columnar (Col_rec_) phase at −5 °C and a columnar phase at −25 °C (Figure 2b, Figure S15). Moreover, a broad scattering reflection around 9 nm^−1^ is visible in the transmission scattering profile representative for the amorphous siloxane halo. It indicates that well‐defined nanoscale morphologies are induced by the self‐aggregation of the *o*DMS moieties in the solid state. In addition, the appearance of sharp scattering peaks at −25 °C in the wide‐angle region (*q*>7 nm^−1^) suggests the presence of π‐stacking between the azobenzene aromatic moieties.

We prepared LCN films by photopolymerization of a mixture of **RM‐AzoSi_3_** and photo‐initiator (Irgacure 819, <1 wt %) at 0 °C, 25 °C and 40 °C with irradiation by non‐polarized white light using a shorter wavelength (<405 nm) cut filter for 20 minutes. The films obtained were glassy, birefringent and did not show LC phase transitions in the DSC thermogram even up to 200 °C (Figure 2a, Figure S16). Furthermore, in SAXS, the film polymerized at 25 °C exhibited a smectic C′ phase showing that the LC structure is retained after photo‐initiated polymerization (Figure 2b). The films polymerized at 0 °C and 40 °C showed similar results with polymerization into Col_rec_ and Sm C LC phases (Figure S17). Variable‐temperature SAXS measurements also showed that the domain spacing *L*_0_ of the polymerized film did not change over temperature, due to the important cross‐linking density of the polymer network (Figure S18). These results indicate that three‐dimensional structures of the LC phases were successfully trapped into the polymer network by photopolymerization using non‐polarized light.

We then explored the imprinting of photoalignment into the structure of the LCN during its photopolymerization by polarized light at 25 °C. First, we demonstrate the linear photo‐orientation of **RM‐AzoSi_3_** in thin films with linearly polarized light (LPL). The monomeric **RM‐AzoSi_3_** was spin‐cast onto a clean, non‐pretreated glass substrate and illuminated under nitrogen with 405 nm LPL at 25 °C for 5 min to yield a thin glassy film. The 405 nm LPL irradiation has a double role to align the monomers with their dipole moments perpendicular to *E* of the LPL and to initiate the polymerization of the acrylate end‐units of the monomers. Indeed, the LD spectrum of the film obtained exhibits a strong signal centered at 335 nm, indicating the linear orientation of the structures within the film (Figure 3a). In polarized UV/Vis spectroscopy measurements, the LPL‐irradiated thin film showed anisotropic absorbance with a dichroic ratio of 1.34 and an order‐parameter of 0.10 at 350 nm (Figure S19). The maximum absorbance is observed perpendicular to *E* of the LPL, confirming an orthogonal orientation of the azobenzene moieties to *E*.

**Figure 3:**
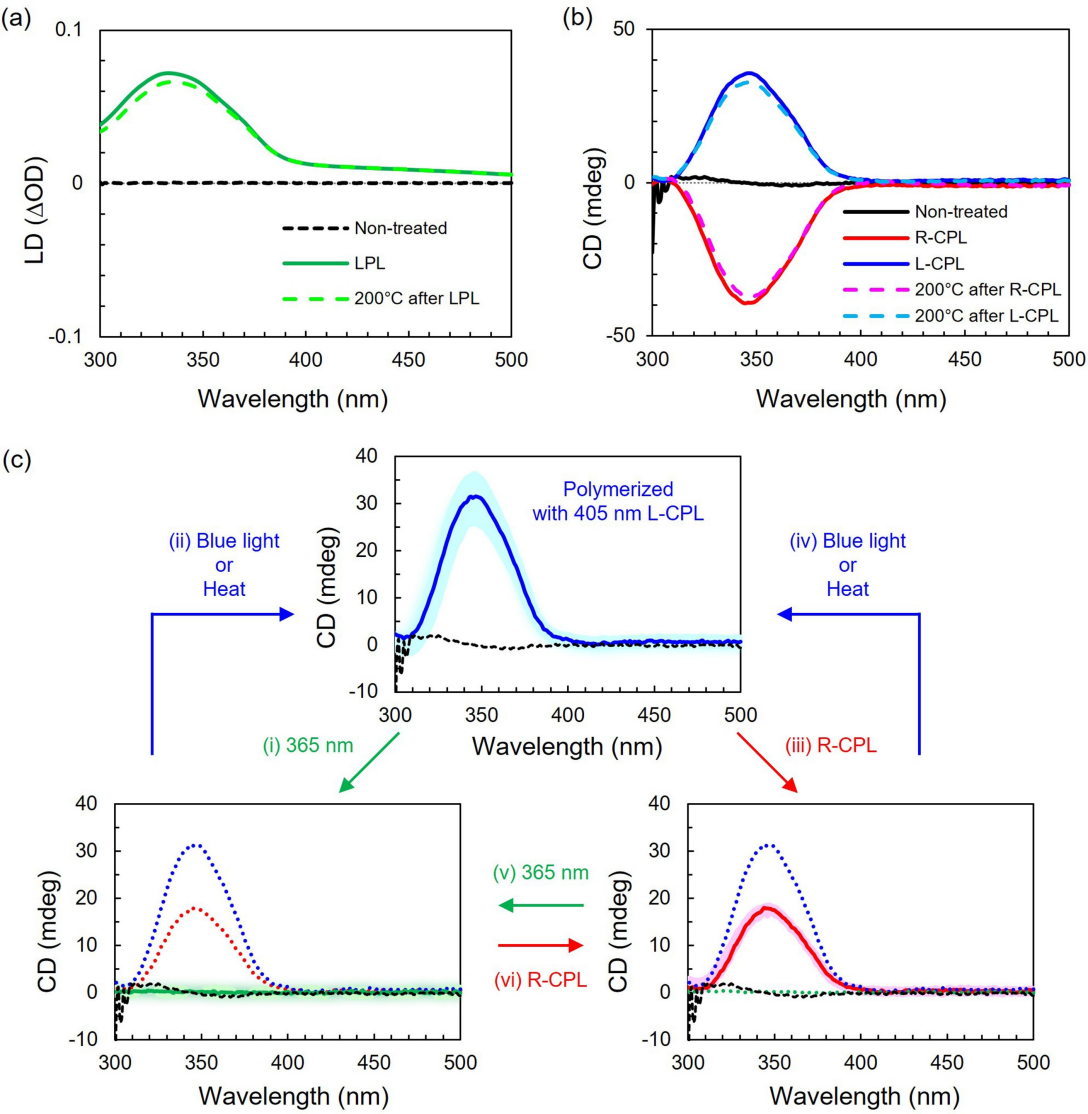
a) LD and b) CD spectra of spin‐cast thin films of **RM‐AzoSi_3_**. LPL and CPL irradiation was carried out with 405 nm LED at 25 °C for 5 min. Heat annealing was carried out at 200 °C for 5 min. c) Changes in CD spectrum of a film photopolymerized with L‐CPL of 405 nm after subsequent steps of photo‐irradiation and/or heat treatment. Black dotted traces indicate a film just after spin‐casting. Dotted red and blue traces of the initial samples are added to facilitate comparison. Experimental details are in Supporting Information.

We continued with CPL irradiation to imprint helical organization within the LCN during its polymerization. Initially, spin‐cast thin coatings of achiral **RM‐AzoSi_3_** were CD silent (Figure 3b). They were irradiated with 405 nm L‐CPL at 25 °C for 5 min. The thin films obtained display a positive CD signal of about 35 mdeg at 346 nm and no LD signal, suggesting a chiral supramolecular structure. Irradiation with R‐CPL gave the mirror image CD signal, confirming that opposite CPL produced enantiomeric supramolecular structures. The dissymmetry factor for absorption of L‐ and R‐CPL (*g*_abs_=2×(A_L_−A_R_)/(A_L_+A_R_)) in the **RM‐AzoSi_3_** film at 350 nm was about 0.003, showing that similar left‐ and right‐handed structures can be obtained. Moreover, the molecular structures within the LCNs show excellent thermal stability. The LD and CD signals were maintained even after heating to 200 °C, indicating the strong propensity of the LCNs obtained to maintain the order imprinted by light (Figure 3a, b). The *g*_abs_ of CPL‐irradiated **RM‐AzoSi_3_** films was too small to find clear features in grazing‐incidence small‐angle scattering, but based on previous work,[^7^, ^25^] the helix axis orients perpendicular to the surface.

Although thermally very stable, the trapped structures were easily reprogrammed by light irradiation (Figure 3c, Figure S20). A chiral thin film prepared by photopolymerization at 405 nm L‐CPL irradiation was subsequently illuminated with unpolarized 365 nm LED at 25 °C (Figure 3c, step (i)). The CD signal visible at 350 nm disappeared, indicating the trapped helical structure of the azobenzenes can unwind by isomerization of the azobenzene moieties. Presumably, the densely cross‐linked network, including the acrylate main chains, remains the same but only the azobenzene groups rotate, due to the flexibility of the siloxane/alkylene spacers. Remarkable, the CD signal was recovered by irradiating the film with unpolarized blue LED light of 405 nm or 450 nm for 5 min at 25 °C or by heating at 100 °C for 5 min (Figure 3c, step (ii)). These results indicate that the LCN film keeps memory of the initial helical structures, which can be reconstructed after the collapse. Besides, upon irradiation with 405 nm R‐CPL, the CD signal decreased in intensity from 30 mdeg to 18 mdeg, sign of partial helix inversion (Figure 3c, step (iii)). In addition, when the CD silent film obtained by unpolarized 365 nm irradiation was irradiated with 405 nm R‐CPL, the same positive CD signal of 18 mdeg was observed, indicating that the frozen helical structure can be maintained even under CPL irradiation of opposite helical sense (Figure 3c, step (vi)).

To understand the contribution of photoalignment and photopolymerization on the ordering process, we studied the photoalignment without polymerization. No circular anisotropy could be induced in the thin layers of **RM‐AzoSi_3_** irradiated in absence of photo‐initiator, which prevents polymerization. Importantly, the UV spectra recorded showed a decrease in absorption after LPL or CPL irradiation indicating the azobenzenes re‐orient perpendicular to the surface and became inactive to LPL and CPL (Figure S21). This result points out the essential role of the formation of polymer chains to restrict chain mobility in the imprinting of helical arrangement. This result also explains why photo‐helical alignment has been often reported in LC polymers but not with small mesogens.

The formation of cholesteric structures by light makes it possible to use photo‐patterning to imprint domains with opposite helical structures on the same substrate. Indeed, a thin film was irradiated successively with R‐CPL and L‐CPL through a photomask. A positive CD signal of about 40 mdeg was observed in the L‐CPL irradiated area and the mirror image CD signal was observed in the R‐CPL irradiated area, while the unirradiated area was CD silent. This result confirms that two helical structures of opposite sense were successfully fabricated on one glass plate (Figure 4, Figure S22). The polymerized area showed birefringence with micrometer spatial resolution, even at 200 °C, while the unpolymerized area did not. This shows the possibility in fabrication of nano‐patterned film with opposite helical structures by a conventional photo‐patterning method using azobenzene containing liquid crystalline molecules such as **RM‐AzoSi_3_**. The present work is a step forward in the efforts to make light‐controllable chiroptical materials with exciting applications such as complex diffraction gratings.[^32^, ^33^, ^34^]

**Figure 4:**
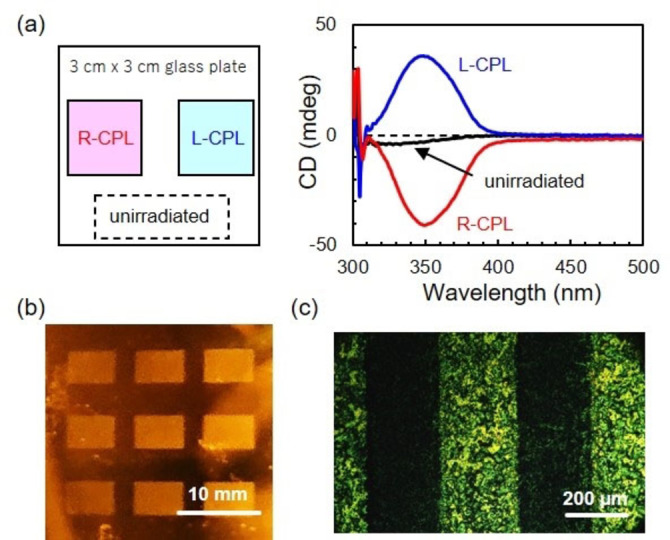
a) Illustration of photo‐patterned spin‐cast **RM‐AzoSi_3_** film and CD spectra of the areas. b) Photograph of a thin film irradiated through a photomask with a rectangular pattern (Figure S22, the bright rectangles are exposed areas; the dark ones are unirradiated areas of non‐polymerized monomers washed away with chloroform for 3 s). c) POM image of a striped pattern at 200 °C (2 μm‐thick film). The unirradiated areas became isotropic and were observed as dark strips.

## Conflict of interest

The authors declare no conflict of interest.

## Supporting information

As a service to our authors and readers, this journal provides supporting information supplied by the authors. Such materials are peer reviewed and may be re‐organized for online delivery, but are not copy‐edited or typeset. Technical support issues arising from supporting information (other than missing files) should be addressed to the authors.

Supporting Information

## Data Availability

All data, materials, and associated protocols that support the findings of this study are shown in the Supporting Information.
