# Supplementary material for: Photo‐Imprinting of the Helical Organization in Liquid‐Crystal Networks Using Achiral Monomers and Circularly Polarized Light
Source: Angew Chem Int Ed Engl. 2022 Feb 18;61(15):e202200839. doi: 10.1002/anie.202200839 (PMC9305743; doi:10.1002/anie.202200839)
Supplement: Supplementary file 1 — Supporting Information [file ANIE-61-0-s001.pdf]

Angewandte Chemie
Eine Zeitschrift der Gesellschaft Deutscher Chemiker

Supporting Information

Photo-Imprinting of the Helical Organization in Liquid-Crystal Networks Using Achiral Monomers and Circularly Polarized Light

*H. Sakaino, D. J. Broer, S. C. J. Meskers, E. W. Meijer, G. Vantomme**

Supporting Information

Table of Contents:

1. Materials, instruments and protocols
2. Synthesis and molecular characterization of **RM-AzoSi$_3$**

1. Materials, instruments and protocols

All chemicals were purchased from commercial sources and used without further purification. Dry solvents were obtained with an MBRAUN solvent purification system (MB-SPS). Oven-dried glassware (120 °C) was used for all reactions carried out under argon atmosphere. Reactions were followed by thin-layer chromatography (TLC) using 60-F254 silica gel plates from Merck and visualized by UV light at 254 nm.

Thin films preparation: Glass slides were cleaned by sonication with ethyl alcohol for 10 min. These glass slides were then etched in a UV-ozone photoreactor (PR-100) for 20 min. To prepare a spin-cast thin film, a solution of the mixture in heptane (3 or 24 mg mL^{-1}) was spin-coated on the etched glass slides (5000 rpm, 40 s, 500 rpm acceleration). The film thicknesses were about 40 or 750 nm, respectively. To prepare the LCN, cells were made by gluing together two glass plates. Glass beads with a diameter of 6 μm were used to precisely control the gap of the cell (except for photo-patterning where beads of 2 μm were used). The cells were filled at the isotropic temperature of the mixture (around 70 °C) and polymerized in their LC phase with UV light source (EXFO Omincure-S2000) using a shorter wavelength (< 405 nm) cut filter for 20 min or with 405 nm or 450 nm LED (8 ± 1 mW cm^{-2}) for 15-30 min. All thin films were stored in the dark and protected by aluminum foil.

Photo-alignment of spin-cast thin films: Photo-irradiation was carried out using a Thorlabs DC4104 advanced four-channel LED driver equipped with a DC4100-HUB. The LED driver

was employed under constant current operation. The light intensity was measured using a radiometer RM-12. To achieve LPL, an adjustable linear polarizer was used between the LED and the thin film. The linear polarizer was at 0° with respect to the *s*-direction of the Fresnel rhomb, with *s* the vector normal to the plane of incidence for the internal reflection in the rhomb. CPL was achieved by adjusting the linear polarizer at 45° and 315° with respect to *s*. The handedness of the CPL was confirmed by a polarizer with the known CPL. The light intensity of the 405 nm and 450 nm LED with the linear polarizer and the Fresnel rhomb was 8 ± 1 mW cm^{-2}. A thin film was placed on a hot plate in a box filled with N_2 gas and the film was kept at 25 °C, then the irradiation was started. After the irradiation, the film was removed from the hot plate and was stored in the dark with aluminum foil. LD and CD spectroscopy measurements were performed at 20 °C as soon as possible after the irradiation.

*Reprogrammed thin films by light irradiation (**Figure 3c**):* Steps (i) and (v): UV irradiation with unpolarized 365 nm LED (4 ± 1 mW cm^{-2}) for 5 min at 25 °C. Steps (ii) and (iv): light irradiation with unpolarized 405 nm or 450 nm LED for 5 min at 25 °C or heat treatment for 5 min at 100 °C without light irradiation. Steps (iii) and (vi): R-CPL irradiation with 405 nm LED for 5 min at 25 °C.

Spectroscopy: NMR spectra were recorded on a Varian Mercury Vx 400 MHz (100 MHz for ^{13}C) spectrometer. Chemical shifts are expressed in ppm, and are referred to the residual peak of the solvent. Peak multiplicity is abbreviated as s: singlet; d: doublet, q: quartet; p: pentet; m: multiplet; dd: double doublet; dt: double triplet; ddt: double doublet of triplets. Matrix assisted laser absorption/ionization-time of flight mass spectra (MALDI-TOF) were obtained on a PerSeptive Biosystems Voyager DE-PRO spectrometer using α-cyano-4-hydroxycinnamic acid (CHCA) or trans-2-[3-(4-tert-butylphenyl)-2-methyl-2-propenylidene]-malononitrile (DCBT) as matrix. CD spectra, LD spectra and UV-Vis spectra of polymer thin films were measured on a JASCO J-815 CD Spectrometer equipped with a Peltier temperature controller. A scanning rate of 200 nm/min, a bandwidth of 2 nm, a response time of 0.5 s, a data pitch of 1 nm and 3 accumulations were employed. UV-Vis spectra of solutions were recorded on a PerkinElmer Lambda 750 spectrometer. A scanning rate of 200 nm min^{-1}, a bandwidth of 1 nm, a response time of 0.24 s, a data pitch of 1 nm, a data interval of 1 nm, and single accumulation were employed. Baseline correction was carried out by measuring the transmission of cleaned quartz cells filled with solvents.

Polarized Optical Microscopy: POM was carried out using Nikon Eclipse Ci POL equipped with a Linkam LTS 420 heating stage.

Small Angle X-Ray Scattering: SAXS of thin films were performed on an instrument from Ganesha Lab. The flight tube and sample holder were all under vacuum in a single housing, with a GeniX-Cu ultra-low divergence X-ray generator. The source produces X-rays with a wavelength (λ) of 0.154 nm and a flux of 1 × 108 ph s^{-1}. Scattered X-rays were captured on a 2-dimensional Pilatus 300K detector with 487 × 619 pixel resolution. The sample-to-detector distance was 0.084 m (WAXS mode) or 0.48 m (MAXS mode), and the instrument was calibrated with diffraction patterns from silver behenate.

2. Synthesis and molecular characterization of RM-AzoSi$_3$

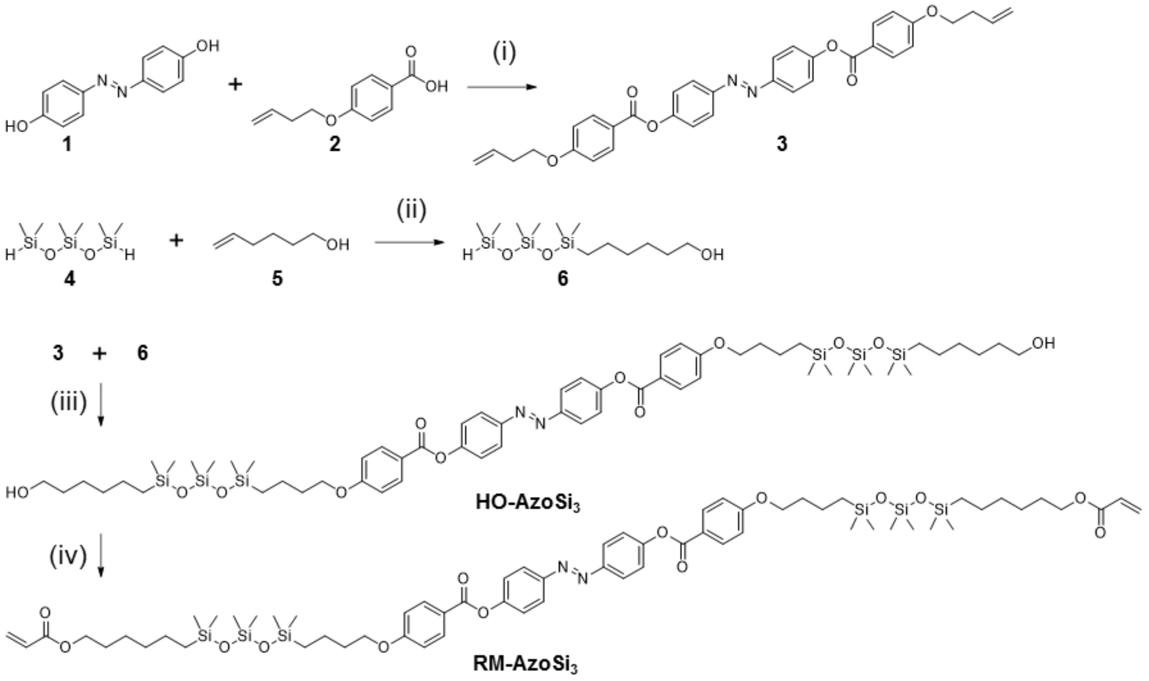

Figure S1. Synthesis route of the azobenzene-siloxane reactive mesogen **RM-AzoSi$_3$**. Reaction conditions: (i) TBTU, TEA, DMF, 60 °C; (ii) Karstedt's catalyst, DCM, RT.; (iii) Karstedt's catalyst, DCM, RT.; (iv) Acryloyl chloride, TEA, DCM, from 0 °C to RT.

*Synthesis of diazene-1,2-diylbis(4,1-phenylene) bis(4-(but-3-en-1-yloxy)benzoate) (**3**):*

A 250 mL dried two-neck round-bottom flask equipped with a magnetic stirring bar under a positive pressure of argon was charged with 4,4'-dihydroxyazobenzene (2.14 g, 10.0 mmol) (**1**), 4-(3-butenyloxy)benzoic acid (4.23 g, 22.0 mmol) (**2**) and N,N-dimethylformamide (60 mL, dehydrated). The solution was stirred at room temperature and dehydrated trimethylamine (4.45

g, 44.0 mmol) was added. After stirring for 0.5 h at room temperature, 1-[bis(dimethylamino)methylene]-1H-benzotriazolium 3-oxide tetrafluoroborate (9.18 g, 28.6 mmol) was added. Subsequently, the solution was heated to 60 °C and stirred for 16.5 h. The crude mixture was cooled to room temperature and water (150 mL) was added. The resulting mixture were filtered and an orange solid was obtained. The resulting solid was washed with hot methanol (150 mL), purified by silica gel column chromatography (R_f = 0.4, chloroform) and washed again hot methanol (50 mL). After drying in vacuo, the pure product was obtained as an orange solid (2.95 g, 53%). ^{1}H-NMR (400 MHz, $CDCl_3$): δ = 8.17 (d, J = 8.4 Hz, 4H), 8.01 (d, J = 8.3 Hz, 4H), 7.38 (d, J = 8.3 Hz, 4H), 7.00 (d, J = 8.4 Hz, 4H), 5.93 (ddt, J = 16.9, 9.9, 6.7 Hz, 2H), 5.18 (dd, J = 22.4, 13.7 Hz, 4H), 4.12 (t, J = 6.7 Hz, 4H), 2.60 (q, J = 6.7 Hz, 4H) ppm; ^{13}C-NMR (100 MHz, $CDCl_3$) δ =164.69, 163.55, 153.29, 150.26, 134.08, 132.52, 124.24, 122.60, 121.60, 117.58, 114.55, 67.62, 33.59 ppm; MALDI-TOF MS (m/z): [M+H]+ calc. for $C_{34}H_{30}N_2O_6$ 563.21, found 563.24.

*Synthesis of 6-(1,1,3,3,5,5-hexamethyltrisiloxaneyl)hexan-1-ol (**6**):*

A 250 mL dried two-neck round-bottom flask equipped with a magnetic stirring bar under a positive pressure of argon was charged with 1,1,3,3,5,5-hexamethyltrisiloxane (2.70 g, 27.0 mmol) (**4**), 5-hexen-1-ol (**5**) (11.3 g, 54.0 mmol) and dichloromethane (27 mL, dehydrated). The solution was stirred at room temperature and xylene solution of platinum(0)-1,3-divinyl-1,1,3,3-tetramethyldisiloxane complex (Pt ~2 %, 135 µL, ~0.27 mmol) was added. After stirring for 30 min at room temperature, the solvent was removed under vacuum and the resulting mixture were purified by silica gel column chromatography (R_f = 0.4, heptane/ethyl acetate=5/1). After drying in vacuo, the pure product was obtained as a colorless oil (2.39 g, 29 %). ^{1}H-NMR (400 MHz, $CDCl_3$): δ = 4.69 (hept, J = 2.5 Hz, 1H), 3.63 (td, J = 6.7, 1.4 Hz, 2H), 1.60 – 1.51 (m, 3H), 1.34 (s, 6H), 0.57 – 0.48 (m, 2H), 0.18 (t, J = 2.0 Hz, 6H), 0.06 (d, J = 1.4 Hz, 10H) ppm; ^{13}C-NMR (100 MHz, $CDCl_3$) δ =63.18, 33.30, 32.87, 25.60, 23.31, 18.32, 1.11 ppm.

*Synthesis of **HO-AzoSi$_3$**:*

A 250 mL dried two-neck round-bottom flask equipped with a magnetic stirring bar under a positive pressure of argon was charged with diazene-1,2-diylbis(4,1-phenylene) bis(4-(but-3-en-1-yloxy)benzoate) (1.41 g, 2.50 mmol) (**3**), 6-(1,1,3,3,5,5-hexamethyltrisiloxaneyl)hexan-1-ol (1.58 g, 5.13 mmol) (**6**) and dichloromethane (150 mL, dehydrated). The solution was stirred at room temperature and xylene solution of platinum(0)-1,3-divinyl-1,1,3,3-

tetramethyldisiloxane complex (Pt ~2 %, 25 μL, ~0.05 mmol) was added. After stirring for 1.5 h at room temperature, the solvent was removed under vacuum and the resulting mixture were purified by silica gel column chromatography (R_f = 0.4, heptane/ethyl acetate=2/1). After drying in vacuo, the pure product was obtained as an orange solid (1.96 g, 66 %). ^{1}H-NMR (400 MHz, $CDCl_3$): δ = 8.17 (d, J = 8.4 Hz, 4H), 8.01 (d, J = 8.3 Hz, 4H), 7.38 (d, J = 8.3 Hz, 4H), 6.99 (d, J = 8.4 Hz, 4H), 4.07 (t, J = 6.4 Hz, 4H), 3.64 (t, J = 6.6 Hz, 4H), 1.87 (p, J = 6.9 Hz, 4H), 1.56 (q, J = 8.7, 7.5 Hz, 9H), 1.35 (s, 11H), 1.25 (s, 2H), 0.67 – 0.58 (m, 4H), 0.54 (t, J = 7.4 Hz, 4H), 0.08 (d, J = 12.7 Hz, 25H) ppm; ^{13}C-NMR (100 MHz, $CDCl_3$) δ = 164.78, 163.87, 153.32, 150.27, 132.51, 124.25, 122.62, 121.36, 114.51, 68.13, 63.24, 33.35, 32.90, 32.73, 25.63, 23.36, 19.91, 18.39, 18.11, 1.48 ppm; MALDI-TOF MS (m/z): [M+Na]+ calc. for $C_{34}H_{30}N_2O_6$ 1201.53, found 1201.53.

Synthesis of ***RM-AzoSi$_3$****:*

A 100 mL dried three-neck round-bottom flask equipped with a magnetic stirring bar under a positive pressure of argon was charged with **HO-AzoSi$_3$** (1.65 g, 1.40 mmol), dichloromethane (21 mL, dehydrated), and triethylamine (0.50 g, 4.90 mmol). The solution was stirred at 0 °C and acryloyl chloride (0.36 g, 3.92 mmol) was added. After stirring for 19 h at room temperature, the solvent was removed under vacuum and the resulting mixture were purified by silica gel column chromatography (R_f = 0.5, heptane/ethyl acetate = 4/1). After drying in vacuo, the pure product was obtained as a waxy orange solid (1.68 g, 93 %). ^{1}H-NMR (400 MHz, $CDCl_3$): δ = 8.17 (d, J = 8.3 Hz, 4H), 8.01 (d, J = 8.3 Hz, 4H), 7.38 (d, J = 8.3 Hz, 4H), 6.99 (d, J = 8.4 Hz, 4H), 6.40 (d, J = 17.3 Hz, 2H), 6.12 (dd, J = 17.4, 10.4 Hz, 2H), 5.81 (d, J = 10.4 Hz, 2H), 4.15 (t, J = 6.8 Hz, 4H), 4.07 (t, J = 6.5 Hz, 4H), 1.87 (q, J = 7.0 Hz, 4H), 1.66 (p, J = 6.9 Hz, 4H), 1.61 – 1.48 (m, 5H), 1.35 (t, J = 5.5 Hz, 12H), 0.67 – 0.58 (m, 4H), 0.54 (t, J = 7.6 Hz, 4H), 0.08 (d, J = 11.7 Hz, 24H) ppm; ^{13}C-NMR (100 MHz, $CDCl_3$) δ = 166.47, 164.76, 163.87, 153.32, 150.27, 132.51, 130.58, 128.78, 124.25, 122.62, 121.36, 114.50, 68.12, 64.86, 33.17, 32.74, 28.72, 25.82, 23.29, 19.92, 18.36, 18.12, 1.48 ppm; MALDI-TOF MS (m/z): [M+Na]+ calc. for $C_{34}H_{30}N_2O_6$ 1309.55, found 1309.59.

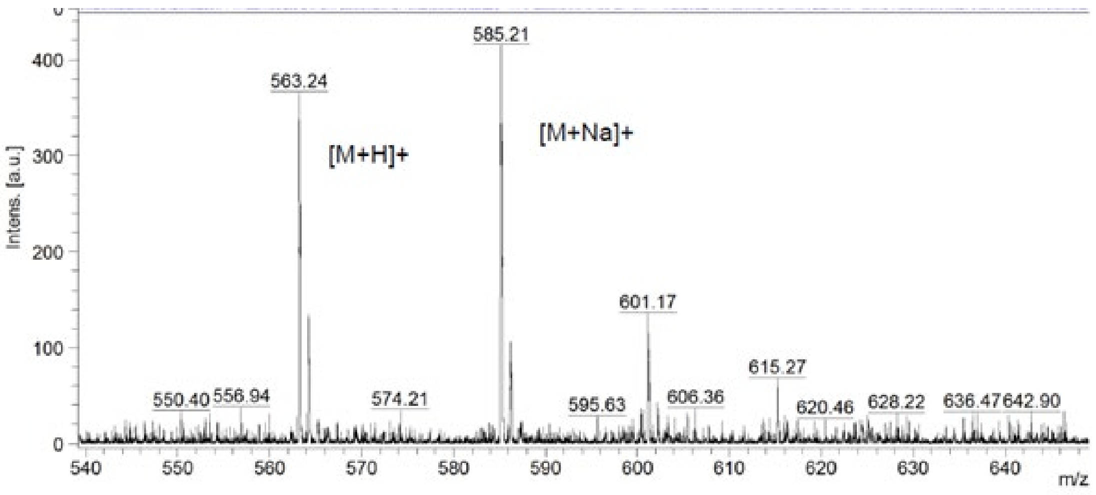

Figure S2. MALDI-TOF Mass spectrum of **3** with DCTB matrix.

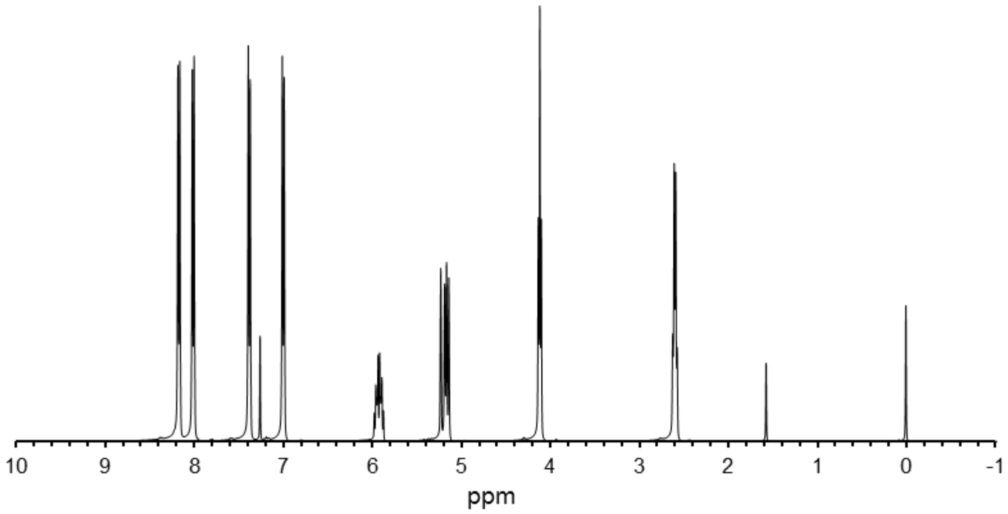

Figure S3. ^{1}H-NMR spectrum (400 MHz, $CDCl_3$) of **3**.

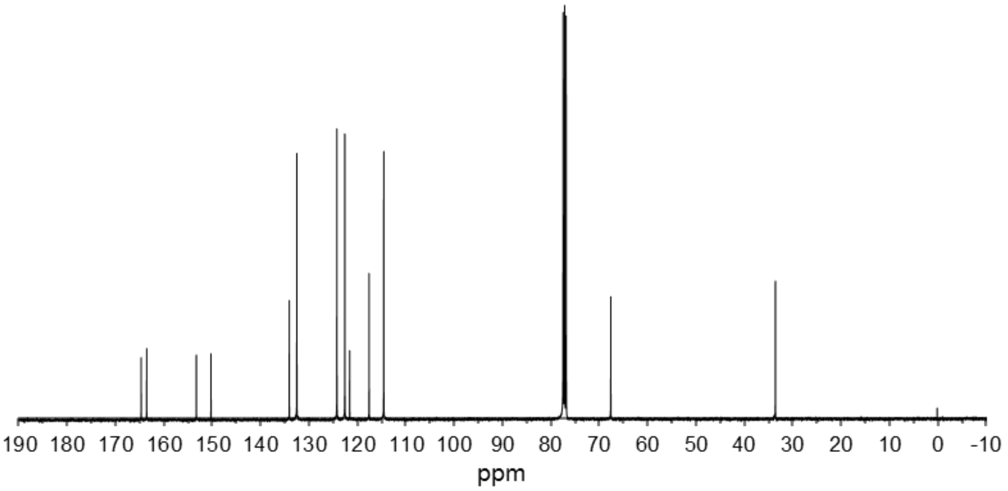

Figure S4. ^{13}C-NMR spectrum (100 MHz, $CDCl_3$) of **3**.

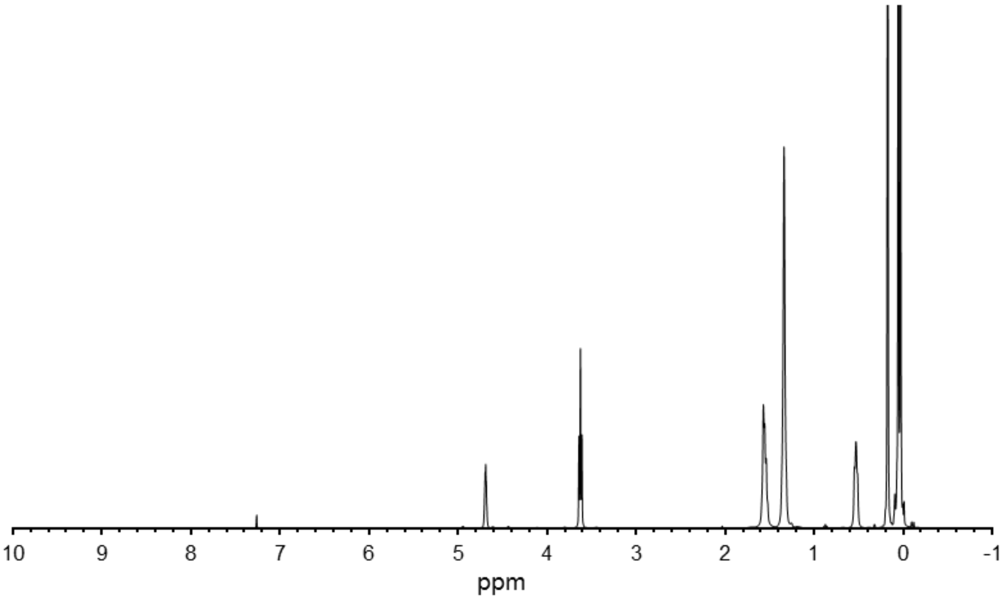

Figure S5. ^{1}H-NMR spectrum (400 MHz, $CDCl_3$) of **6**.

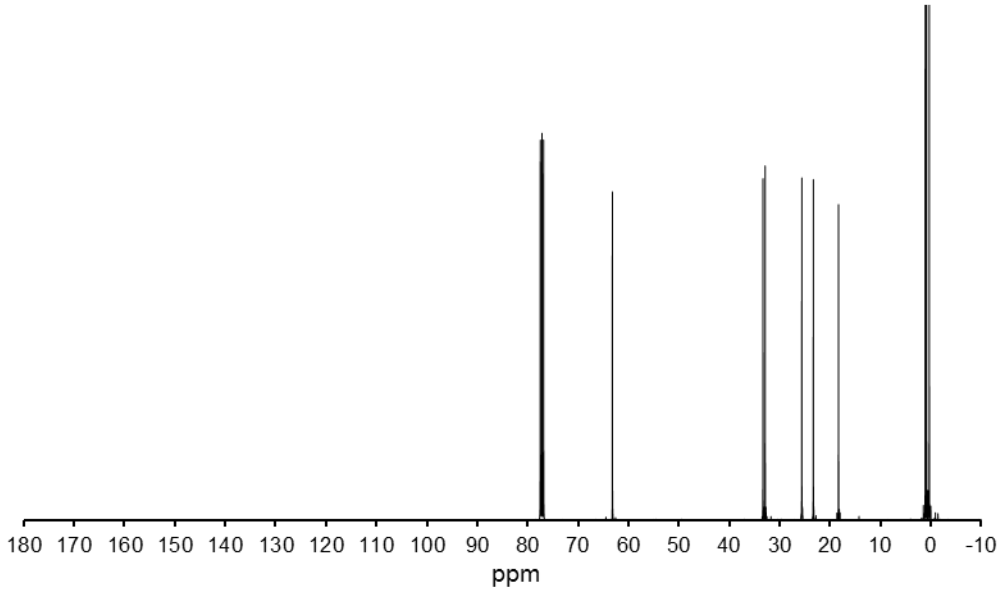

Figure S6. ^{13}C-NMR spectrum (100 MHz, $CDCl_3$) of **6**.

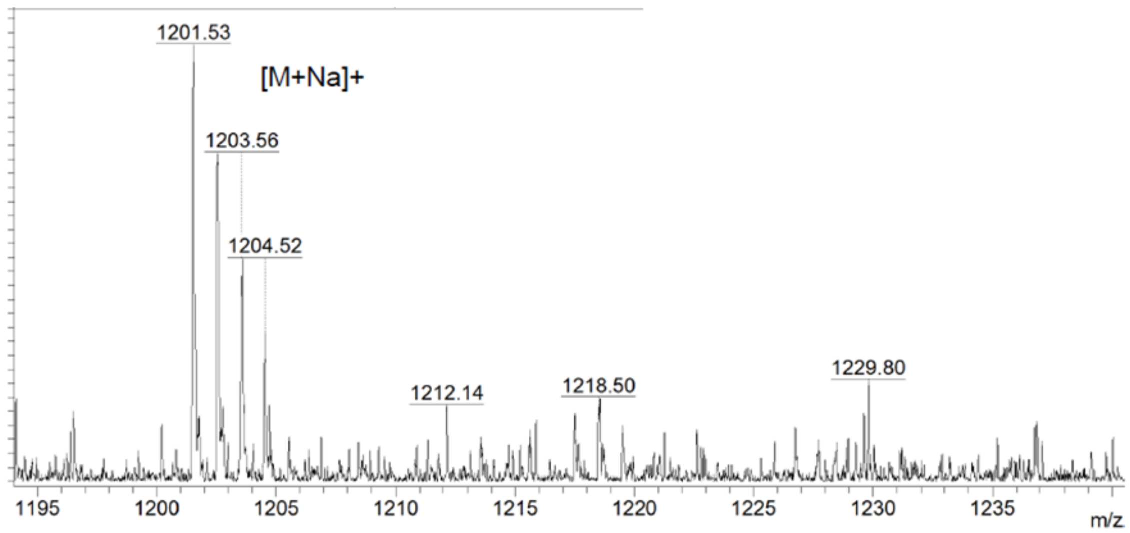

Figure S7. MALDI-TOF Mass spectrum of **HO-AzoSi$_3$** with DCTB matrix.

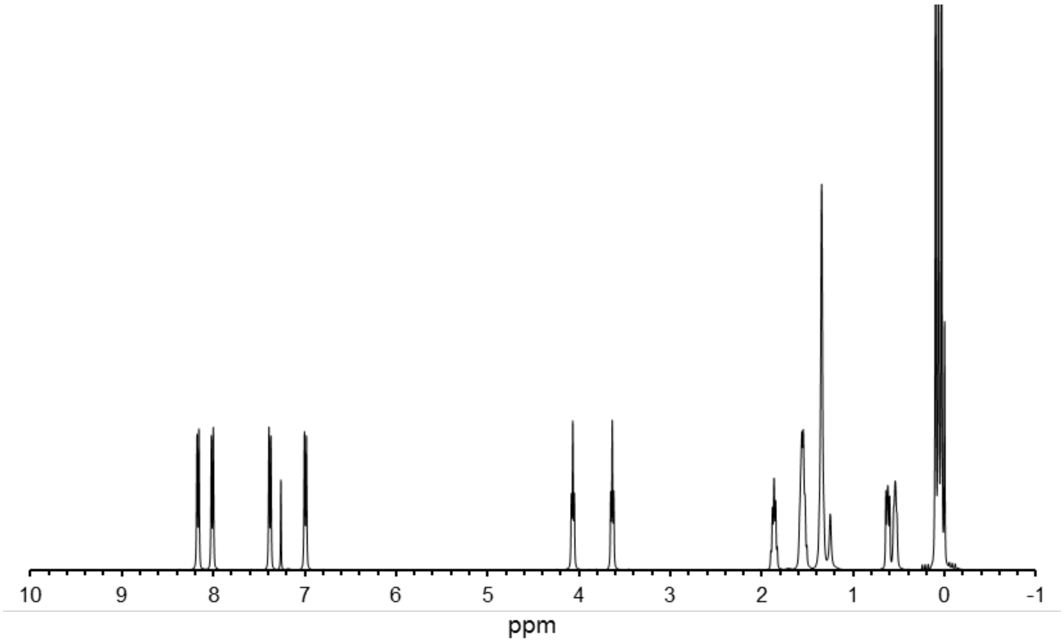

Figure S8. ^{1}H-NMR spectrum (400 MHz, $CDCl_3$) of **HO-AzoSi$_3$**.

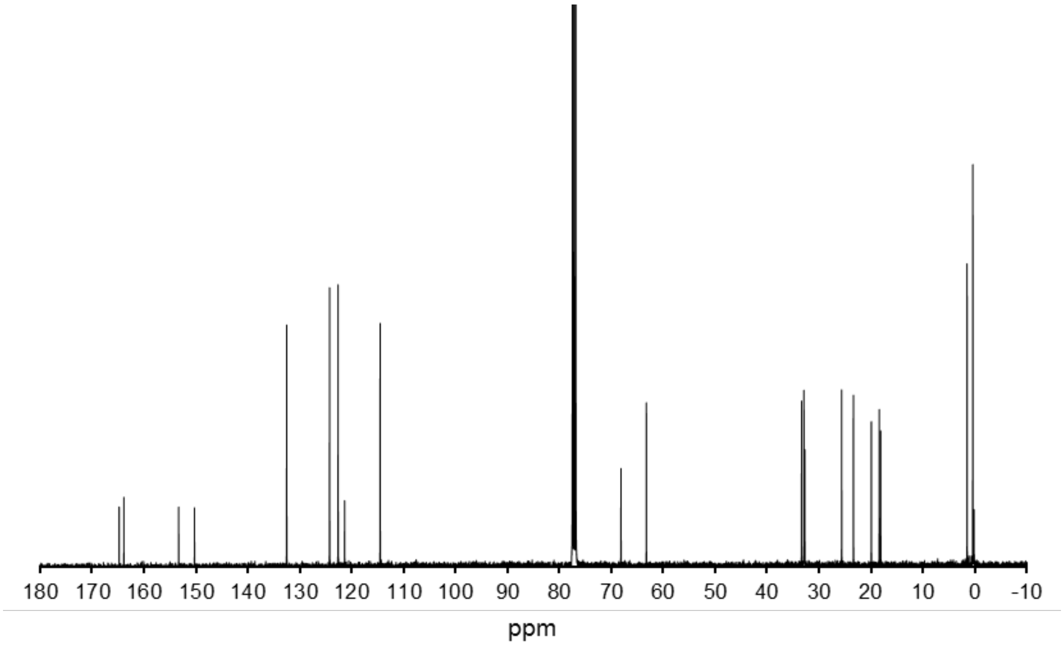

Figure S9. ^{13}C-NMR spectrum (100 MHz, $CDCl_3$) of **HO-AzoSi$_3$**.

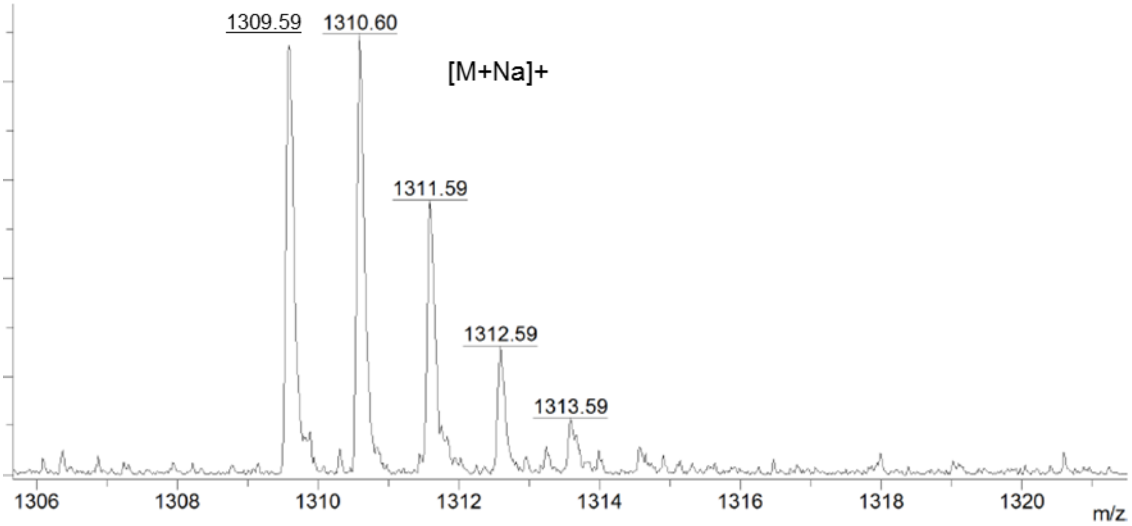

Figure S10. MALDI-TOF Mass spectrum of **RM-AzoSi$_3$** with DCTB matrix.

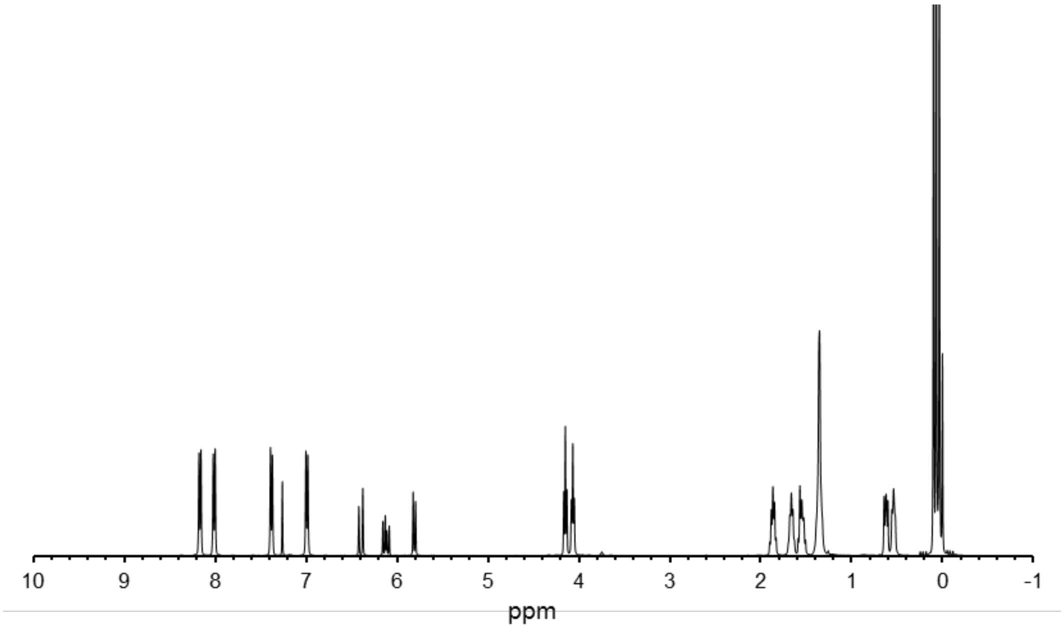

Figure S11. ^{1}H-NMR spectrum (400 MHz, $CDCl_3$) of **RM-AzoSi$_3$**.

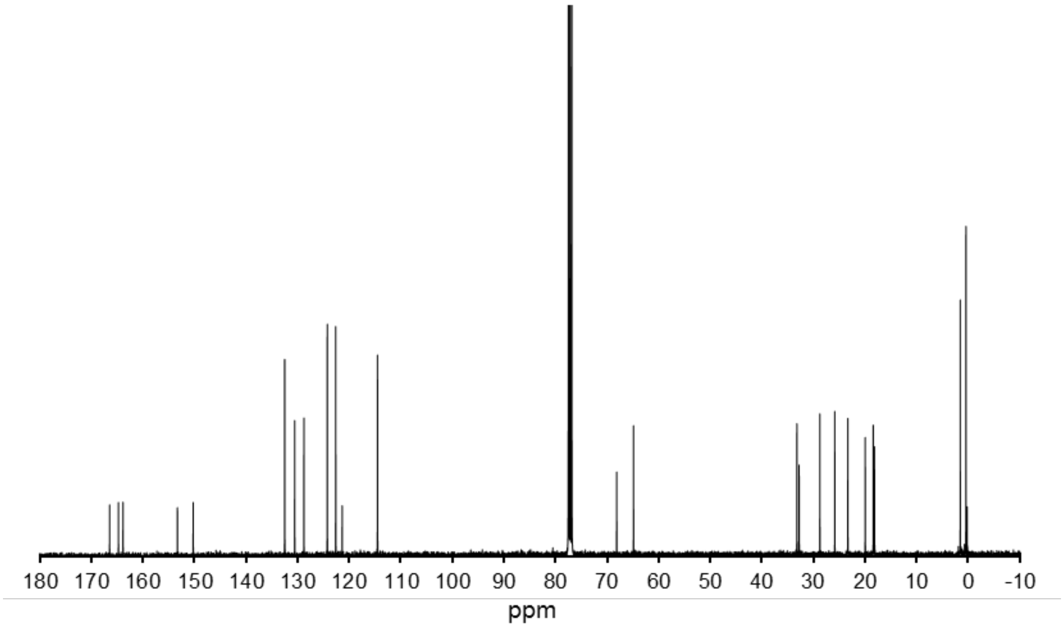

Figure S12. ^{13}C-NMR spectrum (100 MHz, $CDCl_3$) of **RM-AzoSi$_3$**.

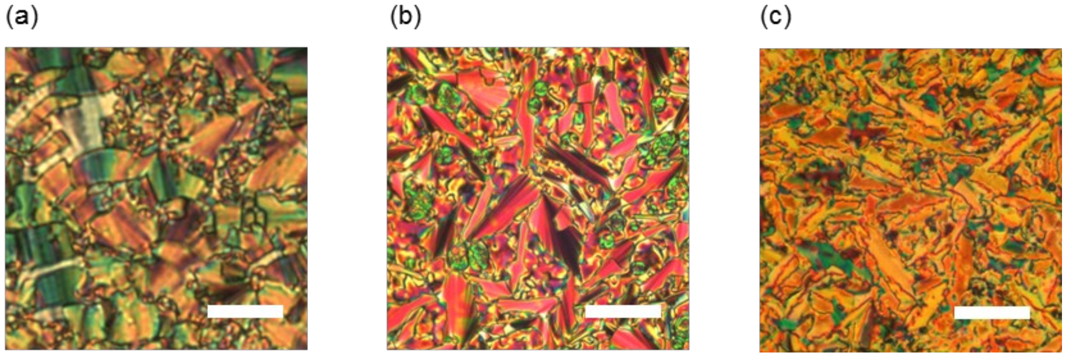

Figure S13. Bulk POM images of **RM-AzoSi$_3$**. Images were captured around (a) 5-10 °C, (b) 20 °C and (c) 40 °C, after the cooling from 100 °C. The study was carried out with polyimide coated glass cells of 6 μm thickness. All scale bars: 100 μm.

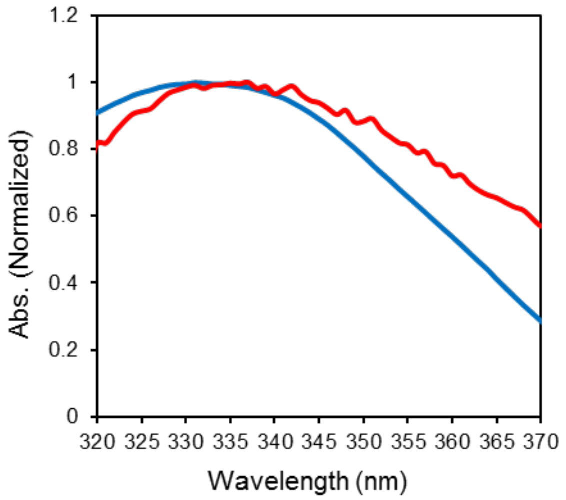

Figure S14. Normalized UV-vis absorption spectra of **RM-AzoSi3**. Blue solid line and red solid line indicate dichloromethane solution (1.0×10^{-5} M) and spin-cast thin film (thickness: ~40 nm), respectively. All the spectra were recorded at 20 °C.

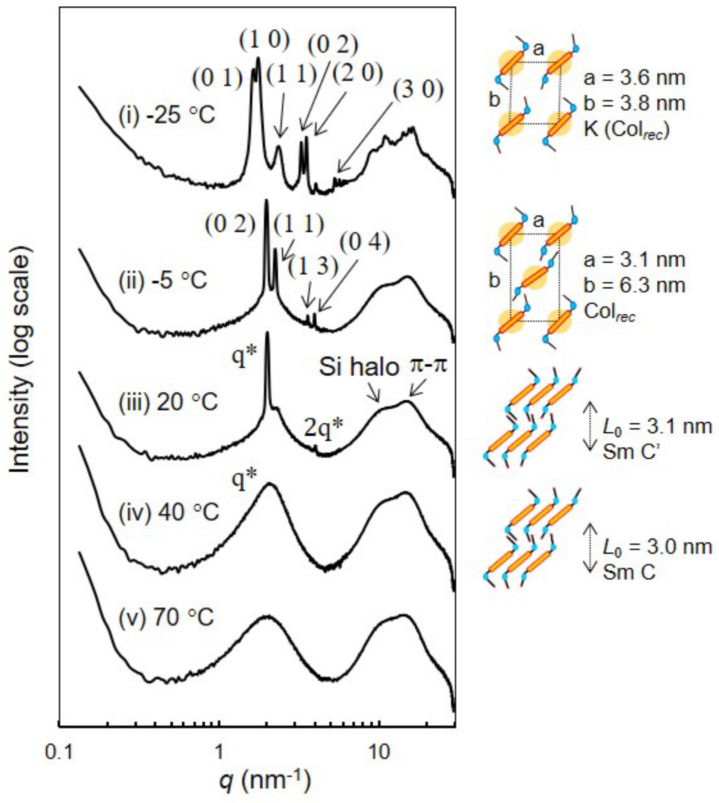

Figure S15. 1D transmission scattering profiles of **RM-AzoSi3** at (i) -25 °C, (ii) -5 °C, (iii) 20 °C, (iv) 40 °C, (v) 70 °C and schematic representations of the molecular packing.

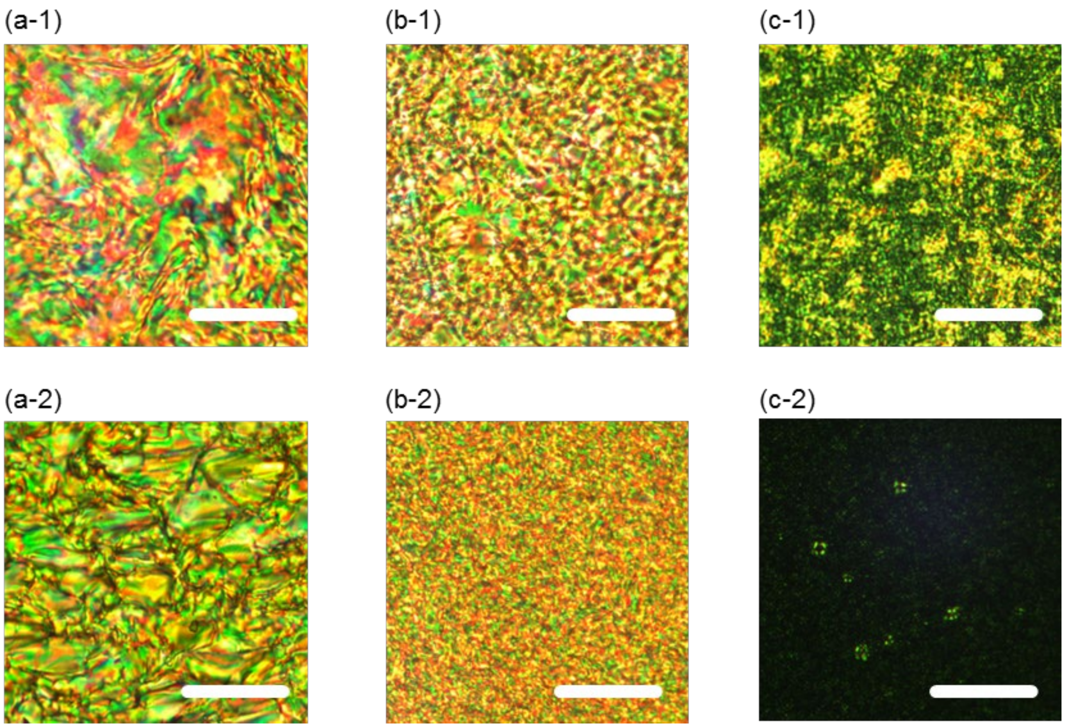

Figure S16. Bulk POM images of **RM-AzoSi$_3$** after polymerization. The polymerization was carried out with UV light source using a shorter wavelength (< 405 nm) cut filter for 20 min at (a) 0-5 °C, (b) 24 °C, (c) 40 °C. The study was carried out with polyimide coated glass cells of 6 µm thickness. (a-1, b-1, c-1) coated with a planar alignment layer (Polyimide Optimer Al 1501, JSR corporation, Japan), (a-2, b-2, c-2) coated with a homeotropic alignment layer (Polyimide Sunever grade 5300). Images were captured at 20 °C. All scale bars: 50 µm.

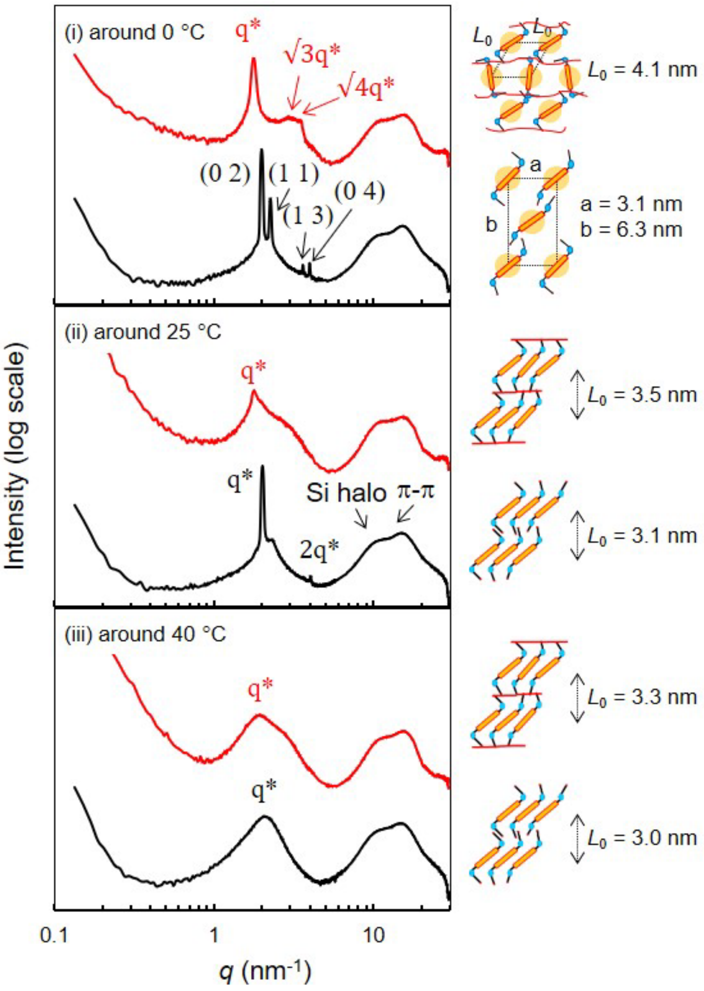

Figure S17. 1D transmission scattering profiles of **RM-AzoSi₃** and schematic representations of the molecular packing. The black line and the red line indicate a bulk at (i) -5 °C, (ii) 25 °C, (iii) 40 °C and a film at room temperature after photo-polymerization at (i) 0 °C, (ii) 25 °C, (iii) 40 °C, respectively.

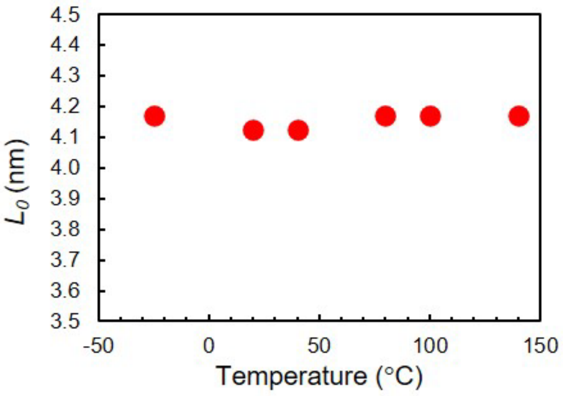

Figure S18. Temperature dependency in the domain spacing L_0 of **RM-AzoSi₃** film polymerized at 0 °C.

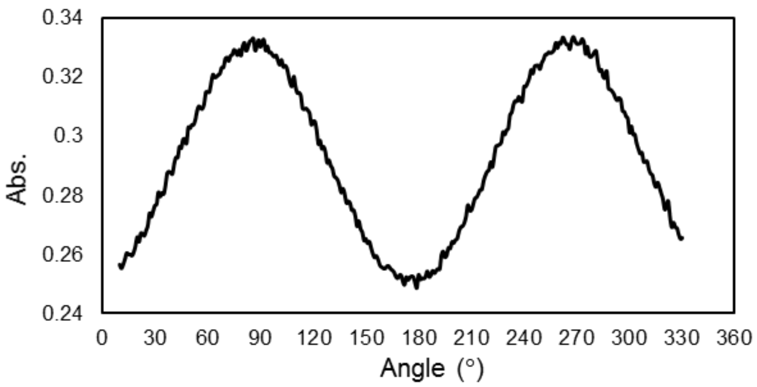

Figure S19. Room-temperature polarized UV-vis spectroscopic spectrum for **RM-AzoSi$_3$** thin film (thickness: ~750 nm) aligned with LPL. The absorbance was measured at 350 nm. The *E* direction of the LPL is 0° and 180°. LPL irradiation was carried out with 405 nm LED (8 ± 1 mW cm^{-2}) at 25 °C for 5 min. The intensity of absorbance at 90° ($A_{\perp}$) was 0.334 and at 180° ($A_{\parallel}$) was 0.249. The dichroic ratio ($A_{\perp}/A_{\parallel}$) was 1.34 and the order-parameter (($A_{\perp}$-$A_{\parallel}$)/($A_{\perp}$+2$A_{\parallel}$)) was 0.10.

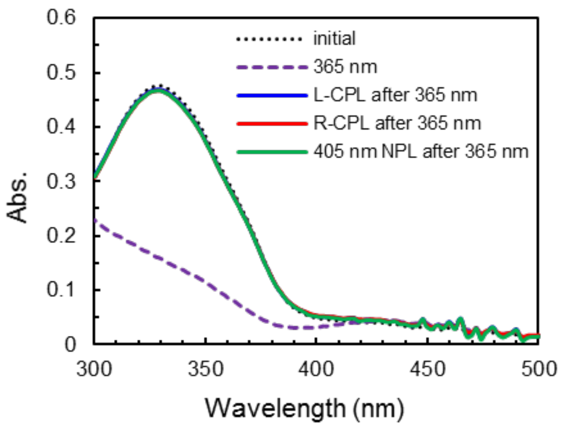

Figure S20. Changes in UV-Vis spectra over multiple steps of photo-irradiation. Black dotted, purple dotted, blue solid, red solid and green solid lines indicate initial film polymerized with L-CPL, 365 nm UV irradiated film, L-CPL irradiated film (after UV irradiation), R-CPL irradiated film (after UV irradiation), L-CPL irradiated film (after UV irradiation), 405 nm unpolarized light irradiated film (after UV irradiation), respectively. The film thicknesses were about 750 nm. UV irradiation was carried out with unpolarized 365 nm LED (4 ± 1 mW cm^{-2}) at 25 °C for 5 min. CPL irradiation was carried out with 405 nm LED (8 ± 1 mW cm^{-2}) at 25 °C for 5 min. 405 nm unpolarized light irradiation was carried out with 405 nm LED (8 ± 1 mW cm^{-2}) at 25 °C for 5 min. All the spectra were recorded at 20 °C.

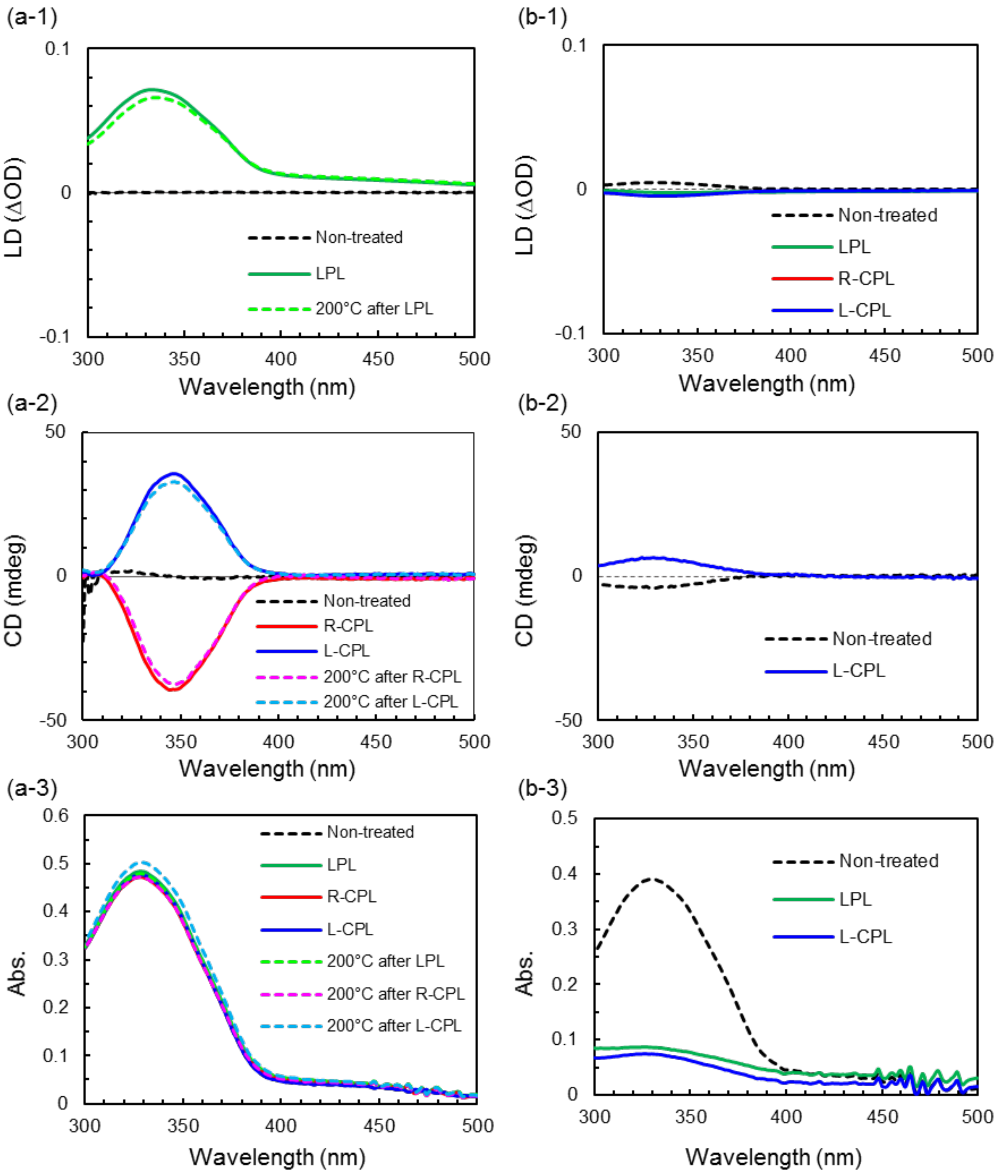

Figure S21. Effects of LPL or CPL irradiation in LD, CD and UV-Vis spectra of spin-cast thin films of **RM-AzoSi$_3$** (a) with a photo-initiator, (b) without a photo-initiator. (a-1, b-1) LD spectra after LPL or CPL irradiation. (a-2, b-2) CD spectra after CPL irradiation. (a-3, b-3) UV-Vis spectra after LPL or CPL irradiation. Black dotted, green solid, red solid, blue solid, light green dotted, pink dotted and light blue dotted lines indicate non-treated film, LPL irradiated film, R-CPL irradiated film, L-CPL irradiated film, heat-annealed LPL irradiated film, heat-annealed R-CPL irradiated film and heat-annealed L-CPL irradiated film, respectively. The film thicknesses were about 750 nm. LPL irradiation and CPL irradiation were carried out with 405

nm LED (8 ± 1 mW cm^{-2}) at 25 °C for 5 min. Heat annealing was carried out at 200 °C for 5 min. All the spectra were recorded at 20 °C.

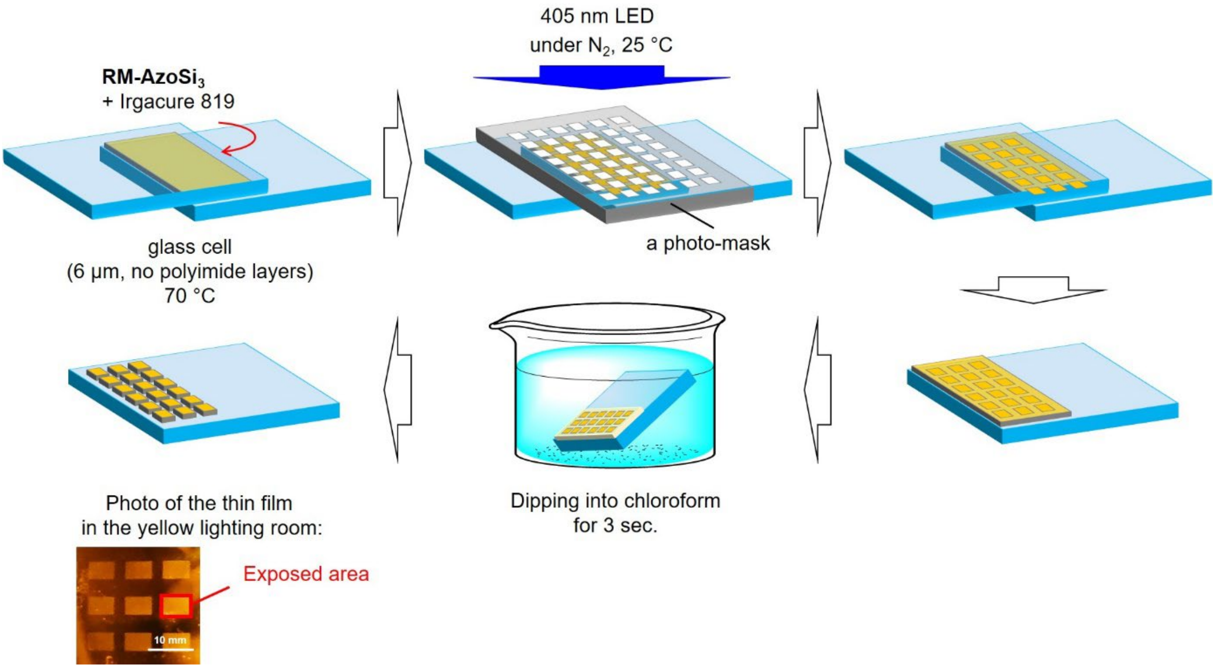

Figure S22. Protocol of the photo-patterning. CPL irradiation was carried out with 405 nm LED at 25 °C for 5 min using a photomask.
